# Wetsuit Thermal Resistivity Measurements

**DOI:** 10.3390/s24144561

**Published:** 2024-07-14

**Authors:** Gianluca Crotti, Roberto Cantù, Stefano Malavasi, Gianluca Gatti, Christian Laurano, Cesare Svelto

**Affiliations:** 1 Dipartimento di Ingegneria Civile e Ambientale (DICA), Politecnico di Milano, Piazza Leonardo da Vinci 32, 20133 Milano, Italy; gianluca.crotti@polimi.it (G.C.); roberto.cantu@polimi.it (R.C.); stefano.malavasi@polimi.it (S.M.); 2 Department of Mechanical, Energy and Management Engineering, University of Calabria, 87036 Rende, Italy; gianluca.gatti@unical.it; 3 Dipartimento di Elettronica, Informazione e Bioingegneria (DEIB), Politecnico di Milano, Piazza Leonardo da Vinci 32, 20133 Milano, Italy; christian.laurano@polimi.it

**Keywords:** wetsuit, material characterization, neoprene, measurement methods, thermal conductivity, thermal resistivity, static and dynamic measurement, thermal transient

## Abstract

In recent years, attention to the realization and characterization of wetsuits for scuba diving and other sea sports or activities has increased. The research has aimed to establish reliable and standardized measurement methods to objectively assess wetsuit quality, particularly focusing on their mechanical and thermal properties. In this work, we describe and compare two different measurement methods for the characterization of neoprene wetsuit thermal resistivity. The first method follows the existing regulations in the field, while the second one, which we are originally proposing in this paper, offers an alternative yet accurate way based on a simplified experimental set-up and easier measurements. In both cases, the wetsuit sample under testing was shaped in the form of a cylindrical sleeve of proper dimensions and wrapped around a phantom containing water at a higher temperature and surrounded by water at a lower temperature. The wetsuit’s cylindrical surface allows heat flow from the warmer water on the inside to the colder water on the outside through the wetsuit area. In the first case, a thermal steady state was achieved, with constant heat flow from the phantom to the exterior. This was obtained with a power balance between two homogenous quantities. Electrically supplied thermal heating within the phantom was used to balance the thermal energy naturally flowing through the wetsuit’s surface. In this first case, a stable and fixed temperature difference was obtained between the inner and the outer surfaces of the wetsuit sample. In the second case, a thermal transient was analyzed during the cooling process of the phantom, and the thermal time constant was measured, providing the sample thermal resistance once the phantom thermal capacity was known. In both cases and methods, the heat flow and thermal resistance of other elements than the wetsuit must be evaluated and compensated for if they are not negligible. Finally, the thermal resistivity per unit area of the wetsuit material was obtained with the product of the wetsuit sample’s thermal resistance and the wetsuit area. The measurements, conducted until now by immersing the phantom in a free surface tank, show that both methods—under stationary and under transient temperature conditions—were valid to assess the wetsuit’s thermal resistivity. The stationary method somehow provided better accuracy while involving less well-known parameters but at the expense of a more complicated experimental set-up and additional energy consumption. The transitory method, on the other hand, is quite easy to implement and, after careful characterization of the phantom’s parameters, it provided similar results to the stationary one. An uncertainty budget was evaluated for both methods, and they did provide highly compatible measurement results, with resistivity values of 0.104(9) m^2^·K/W (stationary method) and 0.095(9) K·m^2^/W (transient method) for the same wetsuit sample under testing, which is also consistent with the values in the literature. We finally propose that the novel method is a valid alternative for characterization of the thermal insulation properties of a scuba diving wetsuit.

## 1. Introduction

The characterization of sports technical materials [1,2] and sportswear [3,4] is important nowadays for research and development in the fields of high-tech sportive tools and fabrics. This is of interest to the industry of producing and selling high-tech materials for sports [5], as well as the end users who will profit from better-quality and more reliable products. In particular, surfing [6] and scuba diving wetsuits [7,8] are made of materials—typically neoprene [9,10]—intended to keep the scuba diver protected from accidental contact in the sea and to keep the human body thermally isolated [11] from the water around it while allowing the maximum freedom of movement in a lightweight and comfortable swimsuit.

Thermal insulation of the human body is particularly important in water, where the heat loss due to convection is much higher than in air. In fact, water’s thermal conductivity (*λ*_w_ ≅ 0.6 Wm^−1^K^−1^ at 20 °C) [12] is much larger than the conductivity of still air (*λ*_a_ ≅ 0.025 Wm^−1^K^−1^) [12]. Thus, it absorbs heat more efficiently from an immersed body, and the liquid temperatures, particularly in cold water, are outside of the range of comfort and balance for humans. Therefore, swimming at the surface and underwater diving in cold water require wearing a thermal insulation suit.

One of the essential properties of a scuba diving wetsuit is its capability to perform effective thermal insulation of the diver’s body in different water conditions in terms of temperature and pressure [10]. Among the other materials, the neoprene rubber in wetsuits is lightweight and flexible because it is manufactured with millions of tiny air bubbles sealed inside the rubber material. Because of this, it provides good wearability, comfort, and thermal insulation. Trapped water between the neoprene and skin quickly reaches the body temperature, and as long as the wetsuit is well worn, the trapped water will show relatively low variation in temperature, resulting in lower convective heat loss. Neoprene has the elastic characteristics of rubber, and thanks to the closed-cell foam of air or nitrogen, it is also a great thermal insulator, providing both fitting and thermal comfort to the body wearing the wetsuit. Foam neoprene [13], made of closed-cell elastomeric foam, is the insulation currently used in the construction of wetsuits. The rubber component of the foam is typically neoprene rubber or polychloroprene, with a thermal conductivity *λ*_r_ = 0.1–0.2 Wm^−1^K^−1^ [14], and the gas component is nitrogen or air, with a conductivity *λ*_g_ = *λ*_a_, thus becoming from 4 to 8 times more insulating than rubber alone. The gas bubbles trapped in the neoprene rubber result in reduced thermal conductivity for the material.

In this work, we present the advancements of a dedicated measurement system [15] and the corresponding experiments and results for the thermal characterization of scuba diving wetsuits. The system is based on the normative requirements in [16] to measure the specific thermal resistance per unit area (thermal resistivity) of a wetsuit under testing, but it also allows for the measurement of a thermal time constant, which can provide a quite good estimate of the same thermal resistivity with a significantly simpler and somehow independent experimental method. Our goal is to propose and validate a novel measurement method as an alternative to the one given by the current norm for rapid and accurate measurements of a wetsuit’s thermal resistivity through thermal transient analysis. This paper is organized as follows. In Section 2, the standard requirements are shown, while in Section 3, we present the basic concepts of heat resistance and resistivity per unit area, which are helpful for this case. In Section 4, we discuss the two measurement methods of a wetsuit’s specific thermal resistance per unit area. The first one is based on thermal steady state operation, and the second one, originally proposed in this work, is based on transient operation. In Section 5, we present the working experimental apparatus based on the requirements in [16], which is capable of performing thermal resistivity measurements both in the steady state condition based on a closed-loop operation (by balancing the thermal flow across the wetsuit material with controlled thermal generation inside a wetsuit phantom using an input electrical power to be adjusted each time, depending on the working conditions) as well as in transient conditions based on a much faster open-loop operation (measuring a time constant under a simple, natural, and unsupervised temperature transient evolution). In Section 5, we show, compare, and discuss the experimental results achieved with the two measurement methods, and finally, we also provide an uncertainty budget of the measurements for each of the two methods. Finally, in Section 6, we draw our conclusions.

## 2. State of the Art and Standard Requirements

Since the 1950s, wetsuits have been produced using neoprene sheets, providing thermal insulation clothing for water sports and scuba diving. The thickness of neoprene determines its suitability for thermal protection. In general, a wetsuit thickness of about 3 mm can be adequate for water temperatures above 24 °C, while for water temperatures between 18 °C and 29 °C, a 5 mm neoprene thickness is used. Finally, for temperatures below 18 °C, the thickest wetsuits of up to 7 mm thick are required [17]. Neoprene wetsuits, in addition to assuring good wearability and comfort, must provide proper thermal insulation to the scuba diver undergoing protracted exposure to cold water at different underwater depths. In particular, a larger suit thickness allows greater thermal resistance but, of course, less comfort. Independent of the original thickness at standard surface pressure (*p* = 1 bar ≅ 10^5^ Pa), the neoprene rubber and hence the wetsuit will significantly reduce in volume and thickness with increasing dive depths (i.e., water pressure). The air bubbles in the neoprene material will shrink in volume with increasing pressure, following Boyle’s law of gases. This reduces the wetsuit thickness and its thermal insulation when the water pressure increases at a rate of ~10^4^ Pa/m with increasing dive depths. At sea level’s atmospheric pressure, the suit has its maximum thickness and highest thermal insulation, but increasing the immersion depth will increase the pressure, and hence the wetsuit’s thermal insulation can significantly decrease with the depth of the dive. Since neoprene wetsuits lower their thermal insulation properties when operating at higher water pressures, the thermal resistance of the sample should be measured at different water pressures, corresponding to the possible depths of scuba diving [18]. The scuba diver can be exposed to “cold” water temperatures (5–20 °C) when diving in winter, at high latitudes, or in lake waters. A thick wetsuit (or for the coldest temperatures, also a dry suit) can be chosen in these cases. However, even in typical recreational and most common sea water dives, at tropical or equatorial latitudes, the scuba diver will be exposed, within the same single dive, to significant temperature gradients between the water surface temperature (20–30 °C) and the maximum dive depth temperature (10–20 °C). For example, in the Mediterranean Sea, the thermocline can be crossed by the diver at depths as shallow as 15–40 m below sea level. The neoprene wetsuit must ensure low thermal flow from the “warm” diver body (>35 °C to avoid any hypothermia) to the external “cold” water, particularly at the highest recreational sea water depths (50 m below sea level and a water pressure of 6 × 10^5^ Pa) and lowest water temperatures (~10 °C in salt or fresh waters). Repeatable and accurate measurements of a wetsuit’s thermal insulation [16] coincident with its thermal resistivity (i.e., specific thermal resistance per unit area) are needed to characterize and compare different scuba diving wetsuits made by different producers and with different manufacturing processes. This is of interest to wetsuit-producing companies and, in the end, the final users (the scuba divers), who want to be ensured that a specific wetsuit provides a given thermal resistivity under typical usage conditions.

Based on a wetsuit’s thermal resistivity (depending on the material and thickness), different wetsuits can be categorized into different thermal performance classes [16], as indicated in Table 1. In particular, to assess the class of a wetsuit, it is important to measure its thermal insulation properties on a quantitative and repeatable basis, thus also allowing clear and objective intercomparisons of different wetsuits.

Measurements of a wetsuit’s thermal insulation are precisely prescribed by recent international regulations [16], proposing not only a measurement method but also a specific experimental apparatus and test procedure to be used to assess a wetsuit’s thermal resistivity in water. Unfortunately, this test procedure, which has to be repeated three times, requires rather long preparation times, since it needs to reach a stationary condition which is possibly different for each wetsuit sample. The achievement of a long-lasting stationary state, as required by the norm [16], is not easy to achieve and typically relies on tedious and time-consuming adjustments of the set-up parameters based on trial and error.

## 3. Theory

In this section, we provide a summary of the basic terminology and equations regarding the thermal resistance of a body (e.g., a given experimental phantom), and when the body is exchanging heat through a given material (e.g., a wetsuit) with a given surface, we derive an expression for the material thermal resistivity (i.e., the wetsuit’s thermal resistance per unit area).

### Thermal Resistance and Thermal Resistivity

A brief theoretical and simplified summary of the process and formulas of heat transfer [19] through the surface of a body and the definitions of the body’s thermal resistance and resistivity per unit area are reported here to help understand and clarify the terms used hereafter. When a temperature difference exists between two bodies or even two parts of the same body, a heat transfer process takes action, with a net flow of thermal energy from the parts at a higher temperature to the parts at a lower temperature. This flowing thermal energy *E* (J) is also called heat, where *Q* = *E* (J), and the process of (net) heat transfer happens continuously over time as long as the temperature difference persists. Considering the elemental unit of time d*t* (s) and the corresponding elemental heat transfer d*Q* (J), we can find the heat transfer rate as follows:

(1) $$\dot{Q}=\frac{dQ}{dt}=\frac{dE}{dt}\;[J/s\;=\;W]$$

which represents the thermal power being transferred from the higher- to the lower-temperature parts. The total heat transferred over a time interval Δ*t* is the time integral of the thermal power over that time:

(2) $$Q=\int_{0}^{\Delta t}\dot{Q}dt\;[J]$$

The heat flux is defined as the heat transfer rate per unit area such that the average heat flux over a surface area *A* (m^2^) can be expressed by

(3) $$\dot{q}=\frac{\dot{Q}}{A}\;[W/m^{2}]$$

In the case of a uniform plane wall of a surface area *A* and thickness *L*, where the two wall surfaces are kept at constant temperature difference Δ*T* = *T*_2_ − *T*_1_, we are dealing with steady thermal conduction through the wall which is subject to a constant conduction heat flux, given by

(4) $$\dot{q}=\lambda \frac{\Delta T}{L}\;[W/m^{2}]$$

where the coefficient *λ* [(W/m^2^)·(m/K)] = [W/(m·K)] is the thermal conductivity of the material and the direction of the heat flow is from the higher to the lower temperature. This thermal conductivity is a thermophysical property of the material, and in general, it depends on the working temperature, but typically with slight variations when operating in a limited temperature interval.

Absolute thermal resistance is the property of a body’s resistance to heat flow when subject to a given temperature difference Δ*T* with respect to the surroundings, and it is expressed as the ratio of the temperature difference to the heat transfer rate:

(5) $$R_{T}=\frac{\Delta T}{\dot{Q}}=\frac{\Delta T}{A\dot{q}}=\frac{L}{A\lambda }\;[K/W]$$

In a uniform material of a given thickness, the specific thermal resistance per unit of area (also called the thermal insulance or thermal resistivity per unit area) is the property of a material’s ability to resist heat flow when subject to a given temperature difference Δ*T* across a given surface area *A*. It is expressed as the ratio of the temperature difference to the heat flux over a given area:

(6) $$R_{T,A}=\frac{\Delta T}{\dot{q}}=\frac{L}{\lambda }=A\frac{\Delta T}{\dot{Q}}=AR_{T}\;[m^{2}\cdot K/W]$$

In Equations (5) and (6), different equivalent expressions are also indicated after the first equality, which gives the definitions of thermal resistance and thermal resistivity. This is carried out with the idea that the equivalent expressions directly depend on quantities originally known or measured in the experiments, and using such expressions and quantities, one can readily evaluate *R*_T_ and *R*_T,A_ with the experimental parameters.

We note that while the thermal resistance *R*_T_ is a property of the body which also depends on the body’s geometry and composition, the specific thermal resistance per unit of area *R*_T,A_ is a thermophysical property of the material. Both *R*_T_ and *R*_T,A_ can depend on the working conditions and in particular on the working temperature, but typically, they are subject to small variations over a limited working temperature range. Thus, for a given wetsuit material and thickness (in practice and also from the same manufacturer), we can expect quite similar if not identical values for the thermal resistivity in the typical wetsuit operating temperature range. However, different wetsuits produced at different thicknesses and by different manufacturers can provide quite dissimilar values for the specific thermal resistance per unit area and hence distinct capabilities in their practical use for resisting the heat flow from a (warmer) scuba diver’s body to the (colder) surrounding water.

## 4. Measurement Methods

In this section, we discuss two different measurement methods for indirectly measuring the thermal resistivity of a wetsuit sample. A first method following the existent regulations [16] is based on a steady state operation, forcing the two sides of the wetsuit to work at constant temperatures and hence with a constant temperature difference between the inner and outer sides of the neoprene wetsuit material. A second method, originally proposed in this work, is based on a transient operation, where the water on one side of the wetsuit dynamically cools down (from its initial, higher temperature value) toward the temperature of the water on the other side of the wetsuit surface (which keeps a constant, lower temperature value during the measurement). Both methods were experimentally tested using the same experimental phantom covered with the same wetsuit cylindrical sleeve and by using the same measurement instrumentation to evaluate the compatibility of their results.

**Figure 1:**
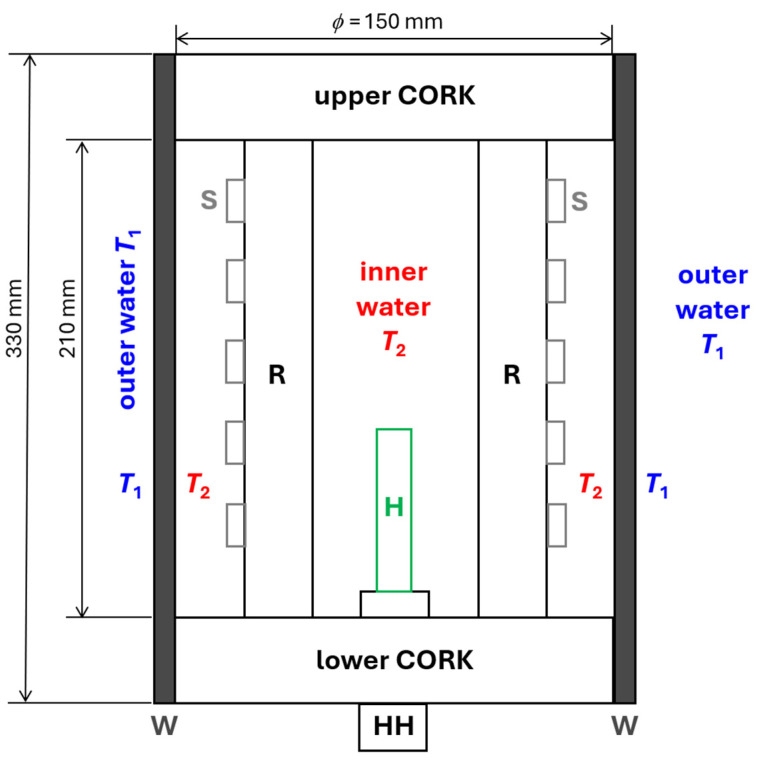
Scheme of the measurement phantom. W = wetsuit; H = electric heater; HH = heater holder; R = rod(s), used as spacers between the lower and upper cork; S = temperature sensor(s).

A schematic drawing of the phantom used for the measurement of *R*_T,A_, based on the indications given in [16], is depicted in Figure 1. The phantom was based on a highly thermal insulating structure (one upper and one lower cork separated by some inner rod spacers) capable of housing one electric heater, an internal water volume, and some temperature sensors enclosed by a cylindrical sleeve of the wetsuit under testing. The phantom was submersed in water, and it exchanged heat mainly through the wetsuit sample between the inner water at temperature *T*_2_ and the outer water at temperature *T*_1_.

Since the scope of this work is to demonstrate the feasibility and substantial equivalence of two different indirect measurement methods, in the following, we will neglect the heat flow passing through the phantom’s corks, which is common for both methods, and we will thus assume that all of the heat flow crossed the wetsuit’s cylindrical sleeve. In fact, even in the presence of non-negligible heat flow through the corks, this common effect on both measurement methods can be compensated for without affecting the intercomparison of the two measurement methods. By naming *R*_T,C_ the thermal resistance of the corks and *R*_T,W_ the one of the wetsuit, the thermal resistance of the phantom *R*_T,P_ (which both methods measure) is then the parallel of the first two resistances. We thus measured the whole-body thermal resistance:

(7) $$R_{T}=R_{T,P}={\left(\frac{1}{R_{T,C}}+\frac{1}{R_{T,W}}\right)}^{-1}$$

Once *R*_T,C_ is known (i.e., one separately measures or theoretically calculates the thermal resistance due to the corks (including their practical and operative differences from perfect Plexiglass cylinders)), the effect of *R*_T,C_ can always be corrected for to obtain from the measured thermal resistance of the phantom—and thus from Equation (7)—the solely thermal resistance of the wetsuit, given by

(8) $$\frac{1}{R_{T,W}}=\frac{1}{R_{T,P}}-\frac{1}{R_{T,C}}$$

In the following, we assume that *R*_T,C_ >> *R*_T,W_, and thus *R*_T,W_ ≅ *R*_T,P_ does not affect the intercomparison of the two measurement methods which, in the presence of smaller and non-negligible *R*_T,C_ values, both underestimate *R*_T,W_ in the same way and by the same amount when using the measured *R*_T,P_ values to estimate the value of *R*_T,W_. Therefore, if both methods estimate the compatible *R*_T,W_ values, even with a non-negligible and unknown *R*_T,C_ value, they both can be used for correct measurement of the real value (Equation (8)) of the wetsuit thermal resistance (*R*_T_ for brevity (and its thermal resistivity *R*_T,A_ = *A*·*R*_T_)) once the cork’s thermal resistance is known and compensated for.

### 4.1. Steady State Operation

In order to characterize the thermal insulation capability of a given wetsuit, the European Standard EN 14225-1 [16] requests evaluating a wetsuit’s thermal insulance (i.e., the specific thermal resistance per unit of area *R*_T,A_ under given test conditions resembling the typical usage conditions of a wetsuit during a scuba dive in cold water). The prescribed testing conditions require performing the measurement in water with two steady temperatures on the two sides of the wetsuit sleeve, namely *T*_2_ ≅ 35 °C (the warmer “inner” or “internal” temperature emulating the human body temperature) on one side of the wetsuit surface and *T*_1_ ≅ 10–15 °C (the colder “outer” or “external” temperature emulating the diving water temperature) on the other side of the wetsuit’s surface. The temperature difference is maintained by injecting a thermal power *P* into the internal water kept at the higher temperature.

In steady state condition, the heat transfer rate across the wetsuit is $\dot{Q}$ = *P* when all the other ways of transfer different from the wetsuit surface are neglected. In particular, the thermal power *P* is supplied by an electric heater (electric resistor supplied with a voltage *U* (V) and current *I* (A)) providing an electric (absorbed) and thermal (generated) power *P* = *U*·*I* equal to the electric power dissipated in the resistor due to the Joule effect. The prescribed wetsuit area experiencing a constant heat flux $\dot{q}$ under the steady temperature difference Δ*T* = *T*_2_ − *T*_1_ is nominally *A* = 0.1 m^2^, and the heat flux is evaluated with Equation (3). 

Finally, the indirectly measured wetsuit thermal resistivity is obtained using Equation (6), which in terms of the experimental parameters and substituting $\dot{Q}=P$ can be rewritten as

(9) $$R_{T,A,\mathrm{ss}}=\frac{\Delta T}{P/A}=A\frac{T_{2}-T_{1}}{U\cdot I}[m^{2}\cdot K/W]$$

where the suffix “ss” recalls the measurement condition under steady state operation.

A disadvantage of the steady state measurement method is that the electric input power has to be finely regulated in order to maintain a stable temperature difference between the two sides of the wetsuit in the prescribed [16] range (Δ*T* = 20–25 °C). The proper value of *P* depends on the specific wetsuit and also on the specific Δ*T* achieved in the experimental conditions. As explained below, we can use two different methods to achieve steady state operation, where by steady state we mean with a “constant Δ*T*” (within a given measurable stability (e.g., Δ*T* < 0.1 °C) over a given time interval of at least 15 min [16]) but in practice with temperatures which slowly change over time *T*_2_ and *T*_1_ and hence a regime which is not completely stationary. In fact, even if the steady state operation keeps a stable Δ*T* value when a given thermal power value (i.e., the electric power *P*) is constantly applied to the phantom, the actual values of *T*_1_ and *T*_2_ will not remain completely stable; both will extremely slowly increase over the long term and not at the same pace. In theory, Equation (6) is valid in a fully stationary regime and thus for both *T*_2_ and *T*_1_ being exactly constant such that *R*_T,A_ undergoes no changes due the material temperature. In practice, as requested in [16], the steady state temperature difference condition should be verified for a long enough time interval (≥15 min, as said earlier), where one should also check that both temperatures *T*_1_ and *T*_2_ are reasonably constant within given small limits, and hence the system is practically stationary.

The first way to achieve electric power regulation is with a closed-loop system, setting and measuring a set Δ*T* value and keeping it stable with an electronic control loop acting on the voltage-by-current product driving the heater. In this way, one automatically regulates the required electrical power value *P* to keep the wanted Δ*T* value, but in practice, *P* can vary over time during a test due to the automatic control loop. If *P* undergoes small variations, then the closed-loop controlled values of *P* and the chosen set point Δ*T* can be both inserted into Equation (9) together with the wetsuit contact area *A* to find the thermal resistivity of the wetsuit sample under testing. This procedure is, however, quite cumbersome and expensive for the usual practice of a typical industrial laboratory performing these thermal measurements, as well as other tests, on commercial wetsuits.

The second way to provide steady state operation while avoiding the use of an electronic control loop, which is more practical and still effective, is to manually adjust the electric power *P* and measure and verify the resulting quasi-steady state Δ*T* condition in the meantime. Of course, when the obtained Δ*T* is not in the prescribed range (Δ*T* = 20–25 °C), one has to readjust the value of *P*, and this requires some manual experience, time, and a good amount of patience. When the achieved Δ*T* value is “stable” (with variations δΔ*T* < 0.1 °C [16], resulting in a relative error *e* = δΔ*T*/Δ*T* < 0.1/20 = 0.5%) and within the prescribed operating range, both values of the manually adjusted *P* and the correspondingly measured Δ*T* are recorded and then inserted into Equation (9) to find the thermal resistance *R*_T,A_ of the wetsuit under testing. Given that a suitable value of *P* is found in a reasonable time (not too long compared with the patience of the operator and the need to not increase *T*_1_ significantly with respect to its initial value), the temperatures can be manually or automatically measured so that an indirect measure of *R*_T,A_ is obtained. This second way of operating is the one that we followed in our experiments. Using a practically large volume of water in an external tank (*V*_T_ > 300 L = 0.3 m^3^, while the water volume within the phantom was *V*_P_~3 L = 3 × 10^−4^ m^3^) with respect to the typical electrical power values (<250 W, even for the lowest resistivity values in Table 1), we could estimate the increase rate of the external tank water temperature to be d*T*_1_/d*t* < 0.43 °C/h. Starting with an initial value *T*_1,MIN_ ≅ 10 °C, the time needed to reach the maximum allowable value *T*_1,MAX_ ≅ 15 °C was a time interval larger than 10 h so that a proper value for the electrical power *P* could be manually adjusted by trial and error in a shorter time. In addition, note that in the experiments with our wetsuit sample operating at 1 bar of pressure, the required electrical power (supplied to the heater) was to the order of roughly 30 W. Therefore, the external water tank temperature could be increasing at a rate d*T*_1_/d*t* < 0.05 °C/h. Anyway, the *P* and Δ*T* adjustment times in this method are not fast, and overall they can last for a few hours or more.

The steady state measurement method, at the expense of the electrical heating system and its power control, being expensive if automatic and tedious if manual, can provide accurate measurements of a wetsuit’s thermal resistance. This happens as long as the phantom’s parameters (mainly the wetsuit surface area *A* when the heat exchange through the phantom’s corks is neglected), the electric power supplied to the heater (and thus the voltage *U* and current *I*), and the measured temperatures (*T*_2_, *T*_1_, and in particular their difference Δ*T* = *T*_2_ − *T*_1_) are properly known. A practical uncertainty budget for this method, in the case of our measurement system, is given in Section 5.4.

### 4.2. Transient Operation

The method which we originally propose for indirect measurement of the thermal resistance of a wetsuit under testing is to perform a test under transient conditions. In this case, the same phantom and temperature sensors described before are used, and a thermal time constant is obtained from an exponential transient behavior. Once again, the temperatures *T*_2_ and *T*_1_ of the water inside and outside of the tank, respectively, are recorded during the process. Also, in this case, the outer water temperature (within the tank) can be set to the “cold” value *T*_1_ anywhere in the range of 10–15 °C (or in practice, and quite simply, one can just let it be stable at an ambient temperature of ~20 °C). This constant cold temperature is the approximate one for the whole volume of the water tank as well as for the outer surface of the wetsuit sample. Instead, the inner water (within the phantom) is initially set to a “warm” value *T*_2,I_ anywhere in the range of 30–45 °C, and this works for the volume of the water and the Plexiglas elements in the phantom as well as the inner surface of the wetsuit sample. Under these conditions, a thermal transient occurs naturally, and the temperature of the inner water cools down to the temperature of the outer water by exchanging heat through the wetsuit surface. Due to the much larger volume and heat capacitance of the water tank with respect to the phantom, during and at the end of the temperature transient, the (outer) water tank temperature *T*_1_ remains substantially unchanged while the (inner) water temperature *T*_2_ within the phantom undergoes an exponential decay over time, given by

(10) T2(t)=[T2,I−T2,F]e−tτ+T2,F

In Equation (10), *t* is the time variable, *T*_2,I_ is the initial value of the inner water temperature at *t* = 0, *T*_2,F_ = *T*_1_, which is the final value of the inner water temperature, and *τ* is the transient time constant. In particular, the time constant *τ* for the phantom temperature transient depends on the phantom thermal capacity *C*_T_ (J/K) and on its (whole body) thermal resistance *R*_T_ (K/W), being simply given by their product *τ* = *R*_T_*C*_T_ (s). Finally, when knowing the area *A* of the wetsuit surface through which the phantom is cooling down to the water tank’s cold temperature, the specific thermal resistance per unit area of the wetsuit sample is evaluated through *R*_T,A_ = *A*·*R*_T_ (m^2^·K/W), as given by Equation (6).

In practical measurements with our set-up and the transient operation method, we had to deal with constant time values ranging from a few minutes up to a few hours (depending on the possible values of *R*_T,A_ = 0.01–0.15 m^2^·K/W for the wetsuits under testing; see Table 1). Therefore, it was relatively simple to record a large number of pairs of temperature and time values *T*_2,*k*_ and *t_k_*, respectively, with *T*_2,*k*_ = *T*_2_(*t_k_*) and with a typical time resolution Δ*t* = *t_k_*_+1_ − *t_k_* = 1 s over an observation time interval to the order of a few time constants. From the recorded set of experimental data points, it is straightforward to retrieve the thermal time constant *τ* with best fit least squares regression of the data to the theoretical transient in Equation (10), and ultimately, one can extract the indirect measure of the thermal resistivity:

(11) $$R_{T,A,\mathrm{tr}}=A\frac{\tau }{C_{T}}$$

where the suffix “tr” recalls the measurement conditions under transient operation.

With this method, the electric heater and its power supply are not required, and no manual or automatic adjustment is needed during the experiment. It is sufficient to start from an inner “warm water” initial condition and then record the temperature values during the temperature transient. In addition, just one temperature, *T*_2_(*t*), needs to be measured (automatically and with no need for manual adjustments) over the time of the test, while the water tank temperature *T*_1_ can be measured or observed (even manually) just at the beginning and at the end of the data recording time interval and only to verify that *T*_1_ did not change significantly during the transient of *T*_2_. After achieving some practical experience with this method and the corresponding experimental system, one can just acquire the temperature samples *T*_2_(*t_k_*) and completely “trust” that *T*_1_ remains constant as long as *V*_T_ >> *V*_P_ (a much larger tank volume than the phantom volume).

After describing the experiments and the results in Section 5.1, Section 5.2, Section 5.3 and Section 5.4, the inaccuracies of the transient measurement method and the corresponding uncertainty budget will also be discussed in Section 5.5, where we also provide a comparison of the results of the two different measurement methods: steady state versus transient.

## 5. Experiments

In Section 5.1, we discuss (and show with specific photos) the different elements of the experimental set-up: the phantom used to “wear” the wetsuit sample, the heater for steady state operation, the external water tank, the temperature sensors, and the data acquisition system. In all cases, the cylindrical phantom for measurement, designed and developed following the procedures in [16], was closed by the wetsuit cylindrical sleeve and completely filled with water prior to its immersion inside the water tank. Two calibrated NTC sensors measured the temperatures *T*_2_ and *T*_1_ of the water inside and outside the phantom, respectively, and hence on the two sides of the wetsuit surface. In all of the measurements, the neoprene wetsuit sample used had a nominal specific thermal resistance per unit area *R*_T,A,NOM_ = 0.1 m^2^·K/W, as measured by an independent metrological laboratory for other wetsuits from the same producer (same neoprene material and sample thickness of 5 mm at atmospheric pressure). To operate without using a powerful water chiller, which just recently was made available in our laboratory, the external tank’s water temperature was naturally regulated by the ambient temperature and stabilized by the large water volume *V*_T_ > 300 L, resulting in *T*_1_ ≈ 20–22 °C, as it slightly changed over time.

After describing the experimental set-up and the phantom, we also present the experimental results obtained in the measurements of the wetsuit’s thermal resistivity using each of the two methods described in Section 4.1 and Section 4.2 and Section 5.3 and Section 5.4, respectively. Finally, in Section 5.5, we compare the measurement results achieved and evaluate an uncertainty budget for the two types of measurements.

### 5.1. Experimental Apparatus

**Figure 2:**
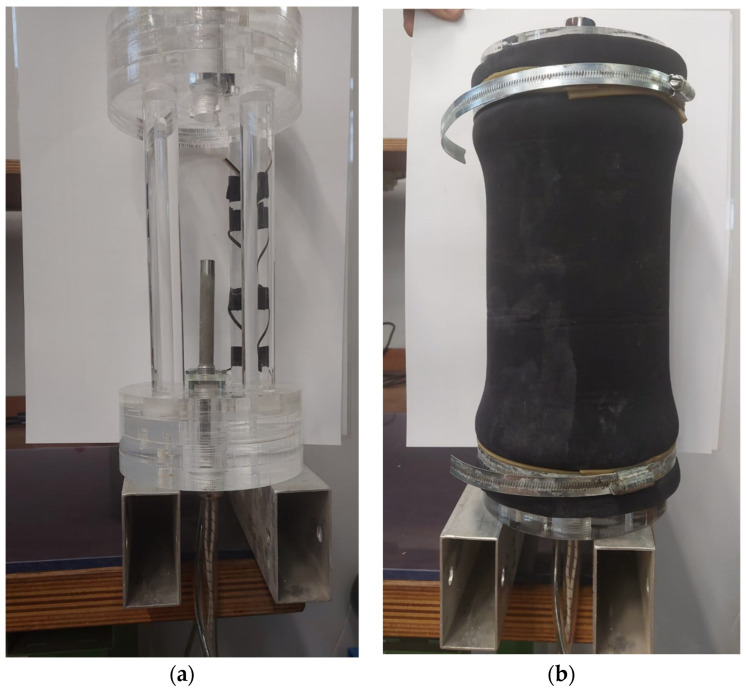
Photos of the measurement phantom. (**a**) The mechanical structure (“naked” phantom) for holding the electric heater, hosting the inner temperature sensor(s), and wearing the cylindrical wetsuit sample. (**b**) The phantom wearing the wetsuit.

In order to measure the wetsuit’s specific thermal resistivity, a custom phantom was realized in agreement with [16] and following the schematic drawing in Figure 1. The material used for construction of the upper and lower corks and for the corresponding four vertical rod spacers was plexiglass (thermal conductivity ~0.2 W/(K·m), specific heat capacity ~1500 J/(K·kg), and mass density ~1.2 kg/dm^3^). Two photos of the phantom, “naked” and wearing the wetsuit’s cylindrical sleeve sample, are shown in Figure 2. Each of the two corks was a Plexiglas cylinder with a diameter of 150 mm and height of 64 mm, while each of the four cylindrical rod spacers had a diameter of 25 mm and height of 210 mm. Note that with such a geometry, the thermal resistance of each cork along the phantom’s axis could be roughly estimated to be (5 m·K/W) × (0.064 m)/(π·(0.15/2)^2^ m^2^) ≅ 18 K/W. With this value for each cork, we obtained a total cork thermal resistance (parallel combination) significantly higher than the phantom’s thermal resistances measured in the experiments (*R*_T,C_ ~ 18/2 = 9 K/W >> *R*_T,P_ ~ 1 K/W), and thus, the assumption of *R*_T,C_ >> *R*_T,W_ could be considered valid. The electric heater was a cylindrical stainless steel rod with a diameter of 12 mm and a height of 100 mm. The heater was commercial with a high-power electric resistance of *R*_H_ ≅ 3.2 Ω, which could be driven by low current and voltage values (*U* ≤ 30 V and *I* ≤ 10 A, respectively) provided by a commercial power supply (RUZIZAO DC power supply, 30 V-10 A). This resulted in measured thermal powers *P* = *U*·*I* up to a maximum value of 280 W, which were applicable to the phantom.

**Figure 3:**
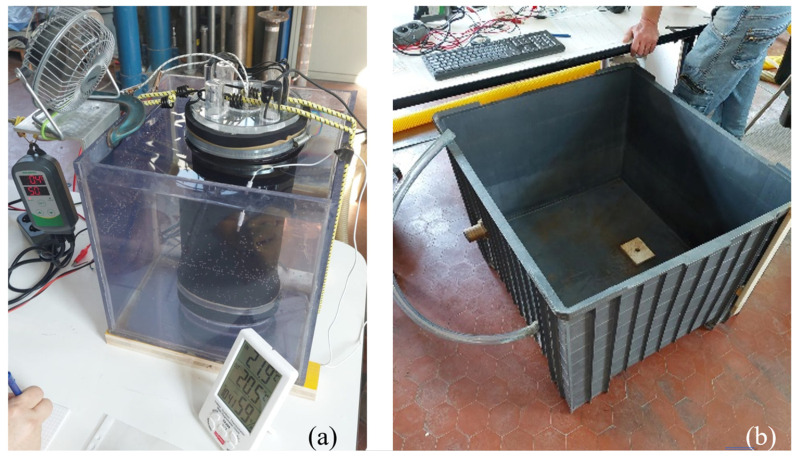
(**a**) Phantom in the smaller water tank. (**b**) Large water tank.

After the phantom was wearing the wetsuit sample, as one can see from Figure 2b, by tightening the wetsuit to the corks by means of cylindrical mechanical clamps, the inner volume of the phantom was filled with water from an upper central hole (with a diameter of 20 mm), which was then hermetically sealed by a stainless steel stopper screw. We note that the inner wetsuit sleeve diameter (*D*_W_ = ~137 mm) for the available wetsuit samples was significantly smaller than the phantom’s cork diameter. This can also be seen in Figure 2b, where the wetsuit was stretched to the larger 150 mm diameter in correspondence to the corks, but it rapidly bent back to the original wetsuit diameter and hence to some ~137 mm along the phantom’s inner lateral wall (see Figure 2b and note the wetsuit shrinking from each cork to the center of the lateral wetsuit area), resulting in a wetsuit area crossed by the heat flux which was significantly smaller than the nominal area (*A* = 0.1 m^2^). The precise volume of water by which the phantom was filled without leaving air bubbles, as observable from the transparent top cork, was measured at the end of the wetsuit test by emptying the phantom’s inner water into a metrically graduated container. Figure 3a shows a photo of the phantom immersed in a smaller and transparent water tank for a preliminary test, and Figure 3b shows a photo of the large water tank (*V*_T_ ≅ 320 L~0.3 m^3^).

### 5.2. Temperature Sensors and Data Acquisition System

**Figure 4:**
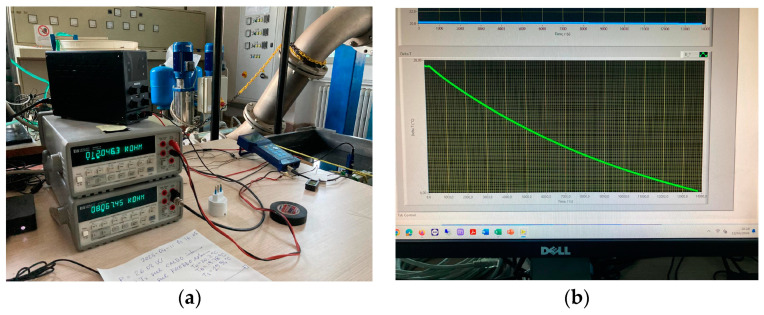
Photos of the temperature acquisition system. (**a**) The DMMs (center left) measuring the resistances *R*_2_(*T*_2_) and *R*_1_(*T*_1_), power supply (on top of the DMMs) used for driving the electric heater, NUC compact PC (bottom left) collecting data from the DMMs, reference thermometer (blue instrument on the table), and water tank (right part of the photo). (**b**) PC monitor showing the plot of a temperature transient *T*_2_(*t*) − *T*_1_(*t*) (green trace, below) and a graph of the constant water tank temperature *T*_1_(*t*) (blue trace, above).

The temperature sensors used to monitor the inner water temperature *T*_2_ (inside the phantom) and the outer water temperature *T*_1_ (inside the water tank) were commercial NTC “10k” thermistors (nominal values *R*_0_ = 10 kΩ at 25 °C and *β*_25/100_ = 3950 K). The thermistor resistance value was read with a precision digital multimeter (DMM; Hewlett-Packard HP3441A, 6½ digits) before exporting the data to a PC via the GPIB digital output using the LabVIEW^TM^ program. Numerical temperature values were read, recorded, and plotted using the PC with a sampling rate of 1 Sa/s. The *R* vs. *T* characteristic of the thermistors used in the measurements was previously calibrated by a precision Pt-100 digital thermometer (DOSTMAN Electronics, P655-LOG), with a rated accuracy of ±0.03 °C over the temperature range from −100 °C to +150 °C. The thermistors were calibrated by obtaining the specific values of *R*_0_ and *β* for each measurement chain, including also the effect of the DMM, including the wires connected to the DDM, and the DMM numerical reading using the data acquisition system (DAS). In the temperature sensor calibration, we measured the resistance values, as read by the DMM and DAS, in correspondence to different temperature values also measured by the reference platinum thermometer. A set of 20 calibration points was used in the temperature range of 10–50 °C. With a nonlinear fitting of the data to the thermistor equation

(12) $$R\left(T\right)=R_{0}\exp\left[-\beta \left(\frac{1}{T}-\frac{1}{T_{0}}\right)\right]$$

with *T*_0_ = 25 °C over the 20 calibration points in the useful temperature range of 10–50 °C, we evaluated the specific values *R_0_ and *β for the calibrated thermistor reading (including the DMM and DAS). These two calibrated parameters were then used for evaluating the measured temperature *T*, obtained by inverting the thermistor equation (Equation (12)), once the electrical resistance *R* was measured in the experiments by the DMM and DAS. In this way, we estimated for our digital and thermistor-based temperature measurement system an uncertainty *u*(*T*) < 0.05 °C in the whole working range of 10–50 °C. Two photos showing the temperature acquisition system are given in Figure 4.

With the described experimental apparatus, we could measure the inner and outer temperatures of the phantom (*T*_2_ and *T*_1_, respectively) while working in steady state or transient conditions. In all of the following experiments, the phantom was closed by the same 5 mm-thick neoprene wetsuit sample, and the tests were carried out at ambient pressure (*p*_ATM_ ≅ 1 bar ≅ 10^5^ Pa) since a stainless steel water tank for performing measurements at a higher water pressure (up to six absolute bars) was not yet available.

### 5.3. Results When Operating in Steady State Condition

**Figure 5:**
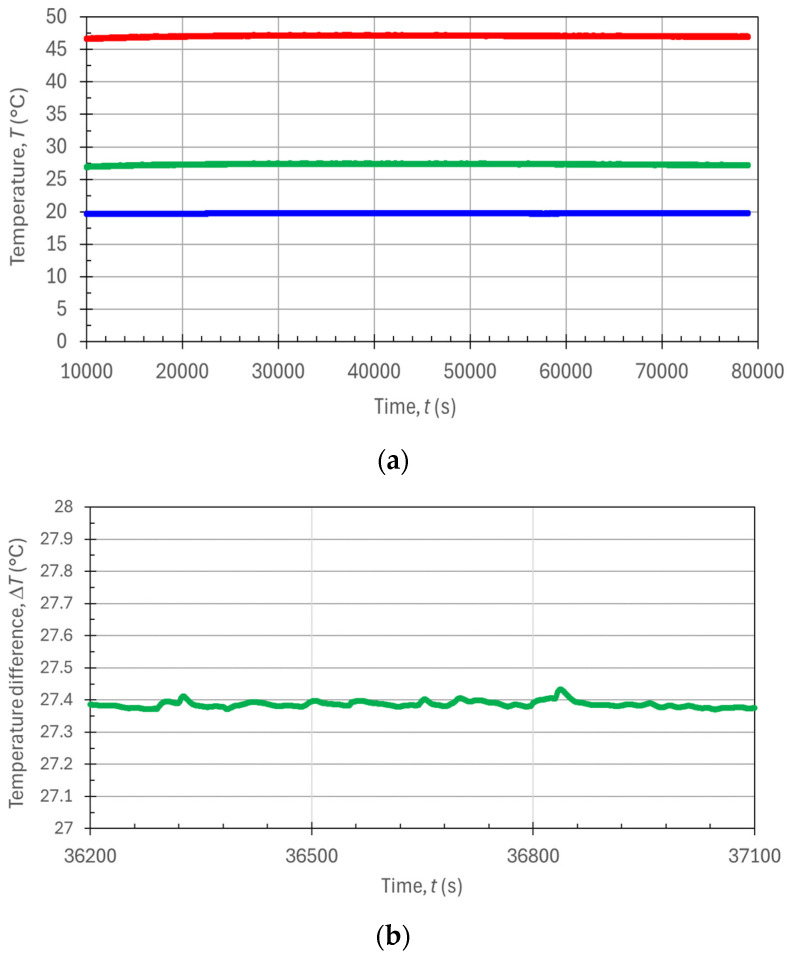
Plots of the measured temperatures as a function of time during the steady state condition: (**a**) temperatures *T*_2_ (red trace), *T*_1_ (blue trace), and Δ*T* = *T*_2_ − *T*_1_ (green trace) and (**b**) zoomed-in section of the Δ*T* vs. *t* plot over a time interval of 900 s.

For the measurements in the steady state condition, the water inside the phantom was heated by the electric heater. By manually regulating the voltage (*U*) and current (*I*) of the power supply driving the electrical resistance, we empirically found that an input power value of *P* = ~30 W for the wetsuit with *R*_T,A_ = ~0.1 m^2^·K/W provided a temperature difference Δ*T* = *T*_2_ − *T*_1_ ≈ 27 °C (thus in the range of 25–30 °C, as prescribed in [16], which can be a typical temperature difference between the human body and the external cold water in recreational scuba diving immersions in cold waters). This experimental condition corresponded to a balance between the ingoing electric power *P*, which is input in a controlled way into the phantom, and the outgoing thermal power $\dot{Q}$, leaving the phantom. Driven by the temperature difference Δ*T*, this thermal power is leaving the phantom toward the external water tank, through the wetsuit’s thermal resistance. In the experiment, when the electrical power level was adjusted so that the temperature difference remained stable within ±0.1 °C for more than 15 min, both temperatures *T*_2_ and *T*_1_ were recorded. Figure 5 shows the results for such steady state measurements obtained with an electric power *P* = 26.08 W, as indicated on the power supply display. We can see from Figure 5a that a rather long initial settling time was needed (Δ*t =* ~10,000 s ≈ 3 h), where *T*_2_ adapted to the manually set electric power value, if one wanted to reach highly stable temperature conditions. After this settling time, both temperatures remained stable—and hence their difference Δ*T* did as well—over an extremely long observation time of ~70,000 s ≈ 20 h. The working regime was practically stationary, and all of the system’s parameters remained substantially constant: *P*, *T*_2_, *T*_1_, and Δ*T*. In particular, Figure 5b shows an expanded view of the temperature difference Δ*T* time diagram, where we can observe temperature stability as good as ±0.05 °C over a time interval of 15 min (900 s), as required by the guidelines in [16].

From the measurement data in Figure 5, and in particular from the values of the stable temperature difference Δ*T* = 27.386 °C (average value over the time interval of 15 min, containing 900 measurement points), upon using the specific value of the electric input power *P* = 26.08 W, one could find the whole-body thermal resistance of the phantom *R*_T_ = Δ*T*/*P* = 1.050 K/W. Then, with the nominal wetsuit surface *A* = 0.1 m^2^, as ideally obtained by the cork circumference (π·150 mm) multiplied by the inner phantom’s height (210 mm), one could find through Equation (6) the measured specific resistance per unit area *R*_T,A,ss_ = *A*·(Δ*T*/*P*) = 0.104 m^2^·K/W, measured in the steady state condition.

### 5.4. Results When Operating in Transient Condition

For the measurements in the transient condition, a water volume larger than the tank’s inner volume was preheated to some higher temperature (*T*_H_ in the range of 40–50 °C) before pouring it into the phantom and sealing the phantom’s upper cork. In this case, there was no need for an electric heater inside the phantom nor precise regulation of its voltage, current, or power values. A few minutes after sealing the phantom, we waited for its thermalization, and the filled warm phantom was immersed inside the water tank which was kept at room temperature (*T*_1_ ≈ 20 °C), which was approximately constant over the whole measurement time. At this point, a temperature transient naturally occurred, with the inner tank’s water temperature *T*_2_(*t*) cooling down from near *T*_H_ (the initial value is not important) down to the regime value *T*_1_. Both temperatures *T*_2_ and *T*_1_ were measured and recorded over time during the cooling process, which is a decreasing exponential given by Equation (10). Since the temperature *T*_1_ remained naturally constant over the measurement, then its value could be measured merely once or twice (e.g., in the beginning and at the end of the transient measurement to check its stability), and thus, only the data points *T*_2_(*t*) had to be recorded by the DAS with the usual sampling period of 1 Sa/s. In fact, for the subsequent fitting procedure used to extract the time constant of the exponential transient, just enough data points of *T*_2_(*t*) were needed, while both values of the initial and final temperatures of the transient were not important, and they could even be rather different from one experiment to the other. The lack of a need for precise values for the temperatures or adjusting to a specific value for the electric power (keeping the temperature difference well monitored) made the transient method far easier and faster than the stationary one.

**Figure 6:**
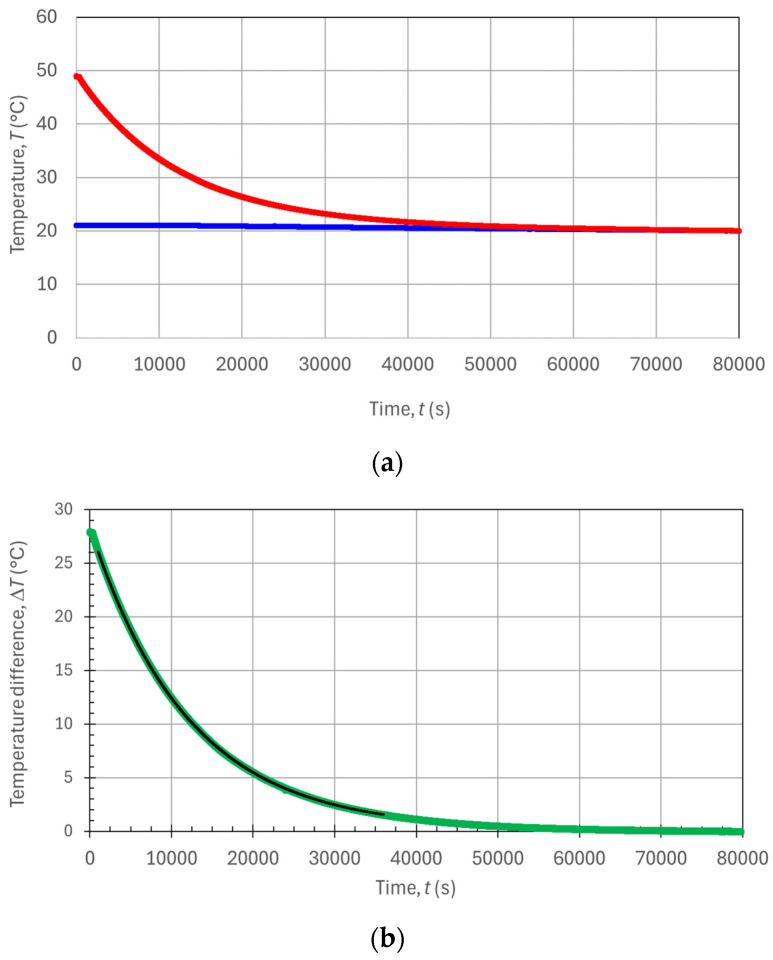

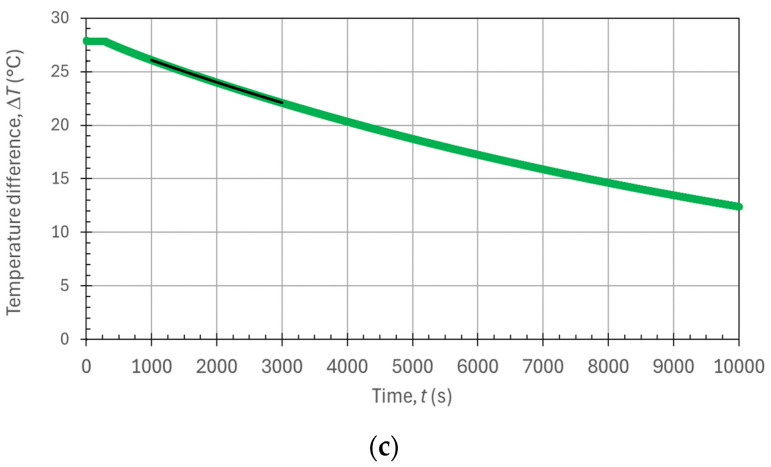
Plots of the measured temperatures as a function of time during the transient condition: (**a**) temperatures *T*_2_ (red trace) and *T*_1_ (blue trace); (**b**) temperature transient Δ*T*(*t*) (green trace) and its fit (black trace) over a 36,000 s time interval; and (**c**) temperature transient Δ*T*(*t*) (green trace) and its fit (black trace) over a 2000 s time interval.

Figure 6 shows the results obtained for the transient regime measurement, with the phantom starting from an initial inner temperature *T*_2,I_ and spontaneously cooling down to *T*_1_ over several hours. In particular, Figure 6a shows the recorded temporal evolution of both temperatures *T*_2_ and *T*_1_ over an extremely long observation time of ~80,000 s ≈ 20 h. This long observation time was indeed recorded only to provide a complete experimental analysis of the temperature’s transient behavior, but it is not needed for ordinary measurements, which can be performed in a much shorter time. Figure 6b,c shows the graphs of the temperature difference evolution Δ*T*(*t*) = *T*_2_ − *T*_1_ corresponding to the experimental points in Figure 6a together with the exponential fitting curve used to extract the time constant *τ* and the variable experimental parameters Δ*T*_I_ and Δ*T*_F_. With reference to Equation (10), Δ*T*_I_ = [*T*_2,I_ − *T*_2,F_] is the initial temperature difference (the transient variable “step”), and Δ*T*_F_ = *T*_2,F_ − *T*_1_ is the final temperature difference (theoretically equal to zero but potentially different from zero due to temperature measurement offsets) at the end of the temperature transient. From the black trace of the exponential fitting curves in Figure 6b,c, we obtained a time constant value *τ* = 12,039.5 s ≅ 12,040 s, evaluated as the average value of *τ*_b_ = 12,028 s from Figure 6b and *τ*_c_ = 12,051 s from Figure 6c.

After measuring the time constant of the phantom natural cooling transient, we could evaluate the phantom’s thermal capacitance *C*_T_ and hence the phantom’s thermal resistance *R*_T_ using Equation (5) to finally obtain the wetsuit’s specific thermal resistance per unit area *R*_T,A_ following Equation (6) (i.e., simply multiplying by the phantom’s area *A*). The volume of the water contained in the phantom when immersed in the water tank was measured both during the phantom’s filling procedure and during the phantom’s emptying procedure, which resulted in *V*_W_ = 3.01 L. The volume of the Plexiglas rods within the phantom was calculated using their geometrical dimensions, resulting in *V*_P_ = 0.41 L, while the volume occupied by the heater was *V*_H_ = 0.011 L.

Considering the specific heat capacity of water *c*_T,W_ = 4180 J/kg·K, we could evaluate the heat capacity of the water in the phantom to be *C*_T,W_ = *m*_W_*V*_W_*c*_T,W_ = *ρ*_W_*V*_W_*c*_T,W_ = 12,582 J/K, where *m*_W_ is the water mass and *ρ*_W_ ≅ 1 kg/L is the water mass density. Similarly, the heater’s thermal capacity (since, for simplicity of operation, the heater was not removed from the phantom during the transient regime measurements) was *C*_T,H_ = *m*_H_*V*_H_*c*_T,H_ = *ρ*_H_*V*_H_*c*_T,H_ = 40 J/K, where *m*_H_ is the heater mass, *ρ*_H_ ≅ 7.7 kg/dm^3^ is the heater’s mass density, and *c*_T,W_ = 460 J/kg·K is stainless steel’s specific heat capacity. Neglecting the small heat capacitance value of the heater, and also neglecting the heat exchanged by the Plexiglas rods within the phantom (since their thermal conductivity is much poorer than water’s thermal conductivity), we could estimate a total phantom thermal capacity approximately equal to the one of the contained water (*C*_T_ ≅ *C*_T,W_). By using this thermal capacitance value together with the measured time constant (for example, *τ* = *τ*_20,000_ = 12,084 s), we found the thermal resistance of the phantom (whole body resistance) to be *R*_T_ = *τ*/*C*_T_ = 0.958 K/W. From this value, by once again multiplying it by the nominal wetsuit area *A* = 0.1 m^2^, one could finally obtain the measured specific resistance per unit area *R*_T,A,tr_ = *A*·*R*_T_ = *Aτ*/*C*_T_ = 0.095 K·m^2^/W, as obtained by the transient condition measurement.

We note that in Figure 6b,c, the initial fitting time is *t*_FIT,I_ = 1000. Instead, the final fitting times were *t*_FIT,F,b_ = 37,000 s for Figure 6b and *t*_FIT,F,c_ = 3000 s for Figure 6c. Thus, Figure 6b refers to a data fitting procedure extended over as long as 10 h and 36,000 experimental points, while Figure 6c refers to a much shorter data fitting procedure extended over only 2000 points (i.e., 2000 s). Through the exponential fitting procedure performed by the Excel^TM^ SOLVER function on the experimental data points of Figure 6, we extracted the *τ* parameters *τ*_36,000_ = 12,028 s = *τ*_b_ from the black trace exponential fitting curve in Figure 6b and *τ*_2000_ = 12,051 s = *τ*_c_ from the black trace exponential fitting curve in Figure 6c. The difference in the two time-constant values was negligible (0.2%), considering that the same percentage error on *τ* would reflect an equal percentage error contribution to the indirectly measured thermal resistivity value, as one can see from Equation (11). For comparison, we note that extending the fitting procedure over other much different (and certainly long enough) time intervals also provided quite similar *τ* values (e.g., *τ*_10,000_ = 12,048 s, *τ*_20,000_ = 12,084 s, and *τ*_50,000_ = 12,112 s). Instead, when working with shorter fitting time intervals (and less fitting points), the obtained *τ* values were slightly underestimated. For example, *τ*_1000_ = 11,953 s, *τ*_500_ = 11,862 s, and finally *τ*_100_ = 11,709 s with only 100 fitting points. We can conclude that fitting the exponential model of Equation (10) to a number of experimental points of the temperature transient to the order of 900–3600 points provided negligible fluctuations in the time-constant evaluation. These numbers of points correspond to time intervals from 15 min to 1 h for the temporal analysis of only the temperature *T*_2_, which were quite practical times for the experiments and also for commercial tests on the wetsuits. As a comparison, we can highlight that with the steady state measurement in Figure 5b (to be compared with the black fitting curve in Figure 6c), the time needed for the measurement was ~10 h, compared with ~1 h for the transitory measurement.

We also finally note that by using this transient condition method, only a simple timing accuracy and reasonable linearity in the *T*_2_ temperature measurement were needed. Any reasonable offsets, even one that varied day by day, in the only temperature (*T*_2_) measurement system did not affect the result of *τ*. After the NTC sensor calibration, both the offset and gain errors were made rather small, and their residual values resulted in negligible inaccuracy in the determination of the time constant *τ*, since the temperature range of the *T*_2_ measurement was limited (up to a maximum excursion of roughly 30 °C). Furthermore, the timing accuracy of the DAS was negligible with respect to the data point sampling time of 1 s and with measured values of *τ* far larger than minutes if not hours. An uncertainty budget analysis, together with an intercomparison of the two methods, is performed in Section 5.5.

### 5.5. Results Comparison and Uncertainty Budgets

As one can see from the results achieved in Section 5.3 and Section 5.4, with the stationary condition method and experiment, we found the specific thermal resistance of the wetsuit under testing (*R*_T,A,ss_ = 0.104 m^2^·K/W), while with the transient condition method and experiment, we obtained the specific thermal resistance of the same wetsuit (*R*_T,A,tr_ = 0.095 m^2^·K/W). The difference between the two measurements divided by their average value provided a relative error of 2.3%, and this was also the relative error between the two measured thermal resistance (*R*_T_) values since in both methods, the same area *A* was used for finding the resistance from the resistivity, and vice versa. Such a value for the relative error between the two measurement methods and the experimental setups is quite acceptable for these kinds of thermal resistance and resistivity measurements, since the thermal performance classes of wetsuits (see Table 1) do change from one to the other by steps in the order of 17%, 18%, and 20% of their average value.

As previously highlighted, the steady state condition method is not simple to implement, requiring the availability and use of an electric heater and tedious manual adjustment of the electrical power used to achieve a useful and long-lasting Δ*T* stationary value. The stationary temperature difference value, as previously said, has to be in the limited range of 25–30 °C, and it strongly depends on the wetsuit’s thermal insulation, thus changing when the wetsuit is changed. The measurement can last from a minimum of about one hour if one already approximately knows the correct electrical power value to up to more than 10 h (depending on the time needed to manually adjust the value of the electric power). Most of this long adjustment procedure has to be repeated when the wetsuit sample is changed. Unless a custom electronic control loop is developed to adjust and regulate automatically the heater power *P*, the steady state measurement requires the constant presence and work of an operator for a rather long time. Furthermore, the simultaneous measurement of temperatures both inside (*T*_2_) and outside (*T*_1_) the phantom is needed, and it must be checked that the temperature difference Δ*T* = *T*_2_ − *T*_1_ remains constant to within ±0.1 °C over at least 15 min of observation time.

On the other side, the transitory state method is far simpler to implement, requiring just the automatic acquisition of one temperature transient *T*_2_(*t*), namely one than uses relatively easy numerical post-processing of the data acquired during the temperature transient, where *T*_2_ is naturally cooling down, without operator supervision from an initial (inessential) higher value toward the final value *T*_1_ (constant and, again, inessential) of the water in the outer water tank.

In terms of the measurement accuracy and uncertainty budget [20], both measurement methods are indirect, and they rely on the measurements in Equations (9) and (11), respectively. When considering all the input and output uncertainties expressed in terms of relative uncertainties and assuming uncorrelated input variables, we can write the relative uncertainty of the measured thermal resistivity obtained by the two methods as follows:

(13) $$u_{r}\left(R_{T,A,\mathrm{ss}}\right)=\sqrt{u_{r}^{2}\left(A\right)+u_{r}^{2}\left(\Delta T\right)+u_{r}^{2}\left(U\right)+u_{r}^{2}\left(I\right)}$$

(14) $$u_{r}\left(R_{T,A,\mathrm{tr}}\right)=\sqrt{u_{r}^{2}\left(A\right)+u_{r}^{2}\left(\tau \right)+u_{r}^{2}\left(C_{T}\right)}$$

Clearly, the uncertainty of the area of the wetsuit surface is responsible for the heat exchange process present in both expressions and with the same weight. At present, the wetsuit samples prepared for the experiments in the form of sewed cylindrical sleeves had a significantly smaller diameter (internal diameter *D*_W_ = ~137 mm before putting the wetsuit onto the phantom) than the phantom’s cork diameter (*D*_C_ = 150 mm). Thus, when the wetsuit was put on the phantom, the wetsuit surface was not the one of a simple cylinder with a circular base and constant diameter from the bottom cork up to the top cork. Instead, this cylindrical surface had a variable diameter, and in particular, its inner diameter started from a larger value *D*_MAX_ = *D*_C_ = 150 mm in proximity of the two corks and reduced itself to a significantly smaller value of *D*_MIN_ ≅ *D*_W_ ≅ 137 mm along most of the height of the phantom. The transition from the larger to the smaller wetsuit diameter was not constant all over the phantom’s height, but it happened mostly in the proximity of the corks such that at a few centimeters away from the corks, the wetsuit’s inner diameter was already at a value of ~137 mm. Due to this manufacturing defect of the available wetsuit, we worked with an uncorrected error to the order of −8% with respect to the nominal wetsuit sample area *A* = 0.1 m^2^, prescribed as the inner wetsuit heat exchange area. While waiting for better cut and sewed wetsuit sleeve samples, we can safely assume that the large area uncertainty *u*_r_(*A*) ≈ 8% for the wetsuit area.

Regarding the other input uncertainties of Equation (13), with our calibrated DAS measuring the two stationary temperatures *T*_2_ and *T*_1_, we could safely assume that each temperature was measured with an absolute uncertainty to the order of *u*(*T*) = ~0.2 °C. Now, considering the measurement of *T*_2_ to be uncorrelated with the one for *T*_1_ and a temperature difference to the order of Δ*T* ≅ 27.5 °C, we obtained [20] *u*(Δ*T*) = $\sqrt{2}$*u*(*T*) ≅ 0.28 °C, and hence we had a relative uncertainty of the measurement of the temperature difference of *u*_r_(Δ*T*) = *u*(Δ*T*)/Δ*T* ≅ 1%. Regarding the measurements of the voltage *U* and current *I*, they both were simply measured by the power meter display, with each having an estimated uncertainty of *u*_r_(*U*) ≅ *u*_r_(*I*) = ~2%. Of course, each of these two electrical quantity measurements could be significantly improved in terms of accuracy by using a good quality and already available DMM. However, in this case, when reducing the electrical variables’ uncertainties below a few percent, then the stability and repeatability of the moderate-quality electrical power supply should also be evaluated and taken into account. In the end, by combining the different uncertainties of the inputs, treated as uncorrelated quantities, one could find the uncertainty budget for the steady state operation measurement to be *u*_r_(*R*_T,A,ss_) ≅ 8.5%, with a dominant contribution from the area uncertainty.

Regarding the input uncertainties of Equation (14), we could estimate through the analysis of repeated *τ* measurements obtained upon changing the length of the fitting time interval a relative uncertainty of *u*_r_(*τ*) ≈ 1%, while the thermal capacitance uncertainty could roughly be estimated to be *u*_r_(*C*_T_) ≈ 4%. The resulting uncertainty budget, in the case of the transient operation, yielded *u*_r_(_R,T,tr_) ≅ 9.0%, again significantly determined by an area uncertainty of *u*_r_(*A*) ≅ 8%.

In the discussed preliminary tests on a commercial scuba diving wetsuit sample (thickness: 5 mm), the two measurement methods provided *R*_T,A,ss_ = 0.104(9) m^2^·K/W and *R*_T,A,tr_ = 0.0948(9) m^2^·K/W, with rather few degrees of freedom for both estimates of the standard uncertainties, henceforth expressed with one digit, as indicated in round brackets after the numerical values of *R*_T,A_. With such relatively large uncertainty intervals (≈9% of the measured value), the two measurements were highly compatible with each other. For now, the uncertainty budget of both measurement methods is strongly dominated by the wetsuit’s area uncertainty, which affects both measurements systematically in the same direction. As a next step, it is foreseen that the same measurements will be repeated and the uncertainty budgets re-evaluated when new wetsuit samples become available, with an inner diameter of 150 mm and different thicknesses. Anyway, the methodology and experimental set-up proposed to measure *R*_T,A,ss_ and *R*_T,A,tr_, as well as the respective uncertainty budgets remain valid.

The results achieved were satisfactory, especially in regard to the main scope of this work (i.e., proving that a different measurement method, namely the transient state measurement, is also available and can be used as a valid alternative to the stationary state measurement method proposed in [16]). With both methods, we could measure a 5 mm neoprene wetsuit’s thermal resistivity in a compatible way with what was previously described in the literature, as foam neoprene of this thickness has been shown to have a thermal resistivity of approximately 0.090–0.100 m^2^·K/W [18,21].

## 6. Conclusions

In this work, we investigated the feasibility of an experimental apparatus capable of measuring the thermal resistivity (resistance per unit area) of wetsuits. The system is based on a measurement phantom developed in accordance with the existing norm and can measure the thermal resistivity of a given wetsuit with the steady state operation also prescribed by the norm. In addition, we proposed and experimented with a different method, which is novel in this application to our knowledge, to measure the same wetsuit’s thermal resistivity with a simpler set-up. In this case, the phantom’s thermal resistance, and hence the wetsuit’s thermal resistivity, were obtained through a transient thermal operation, where the internal temperature in the phantom naturally cooled down to the external water tank’s ambient temperature. The proposed transient method allowed measurement results highly compatible with the ones for the steady state method (0.095(9) K·m^2^/W and 0.104(9) m^2^·K/W, respectively), providing easier and faster repeatable measurements.

A suitable calibration of the phantom’s parameters, in particular for the wetsuit area, must be performed to achieve more accurate measurements and strengthen the compatibility result for the two different measurements. Anyway, the equivalence between the two different measurement methods was proven here theoretically as well as experimentally. Both methods will be tested further using a high-pressure water tank (to also measure the wetsuit’s insulation at six bars of absolute water pressure) and with correct and more accurate values for the wetsuit area, the phantom’s thermal capacitance, and if needed for the voltage and current values as well. When our laboratory is ready to perform calibrated measurements of a wetsuit’s thermal resistivity with both methods, an intercomparison with other laboratories involved in these tests will be performed.

## Figures and Tables

**Table 1:** Thermal performance classes of wetsuits.

Thermal Performance Class	Thermal Resistivity in Water at 1 Bar (m^2^·K/W)	Thermal Resistivity in Water at 6 Bar (m^2^·K/W)
A	≥0.15	≥0.03
B	0.10–0.149	≥0.02
C	0.07–0.099	≥0.01
D	0.05–0.069	≥0.01

## Data Availability

The data that support the findings of this study are available from the corresponding author upon reasonable request.
